# APC Promoter Hypermethylation as a Prognostic Marker in Breast Cancer Patients

**DOI:** 10.31557/APJCP.2020.21.12.3627

**Published:** 2020-12

**Authors:** Pensri Saelee, Tanett Pongtheerat

**Affiliations:** 1 *Research Division, National Cancer Institute, Bangkok 10400, Thailand. *; 2 *Unit of Biochemistry, Department of Medical Sciences, Faculty of Science, Rangsit University, Patumthani, Thailand.*

**Keywords:** Breast cancer, APC hypermethylation-MS, PCR, treatment, distant metastasis

## Abstract

**Background:**

Adenomatous polyposis coli (APC) promoter hypermethylation implicated in breast cancer development through Wnt signaling pathway, hypermethylation may result in inactivation of APC expression. This study aimed to investigated whether hypermethylation of APC promoter, the aggressive behavior of breast cancer cells, and correlated them with clinicopathological parameters and survival.

**Methods:**

Sixty-one fresh tissues of breast tumor were evaluated for APC promoter hypermethylation with methylation-specific PCR techniques (MS-PCR) and APC mRNA expression level analysis by quantitative real-time reverse transcription-PCR.

**Results:**

Our results show aberrant APC hypermethylation status was founded in 27 of 61 cases (44%), and significantly associated with chemotherapy treatment (OR= 6.9, 95%CI=1.5-31.01, P = 0.01), distant metastasis (OR = 5.52, 95%CI = 1.27-24.08, P = 0.04) as well as APC methylated status also associated with shorter overall survival than those without (8.4 and 11.0 years respectively, P = 0.02).

**Conclusion:**

The findings indicated hypermethylation of APC promoter may be used as a useful prognostic biomarker in breast cancer patients.

## Introduction

Breast cancer is the most common malignancy to be found in women. In Thailand, the incidence rate of breast cancer is the highest type of cancers affecting woman about 31.4/100,000 population (Imsamran et al., 2018). 

Several genetic alterations are involved in the development of breast cancer. Such as, the cytosines methylation of the CpG islands located in the promoter region of genes, which may result in a decreased gene expression, or silencing tumor suppressor genes (without altering the genetic code) and is found to occur in many cancers, including breast cancer. (Paluszczak et al., 2006; Lin et al., 2001; Saelee et al., 2014, Jin et al., 2001).

The Adenomatous polyposis coli (APC) gene located on chromosome 5q21-q22 encodes a large multidomain protein, which contains genetic code for 2,843 amino acids, which play a major role in the Wnt signaling pathway, cell cycle regulation, cell differentiation and proliferation (Shen et al., 2016; Han et al., 2018). The APC tumor suppressor gene is involved for familial adenomatous polyposis (FAP) and initiates carcinogenesis for various cancer types (Kinzler et al., 1996; Shen et al., 2016; Han et al., 2018) such as colo-rectal cancer, prostate cancer, gastric cancer and breast cancer (Matthaios et al., 2016; Richiardi et al., 2009; Tsuchiya et al., 2000; Hiltunen et al., 1997; Saelee et al., 2014; Jin et al., 2001).

Thus, we identify the role of APC CpG-island hypermethylation to be a significant factor in Thai breast cancer patients. Sixty-one breast tumor samples, were evaluated by methylation-specific PCR, to detect promoter hypermethylation of the APC gene. Altered APC mRNA expression level were quantified by quantitative real-time reverse transcription-PCR as well. The results of this research may be used to assess the correlation of this gene and the clinicopathological characteristics of breast cancer among Thai women patients and to clarify the appropriate prognostic biomarker for breast cancer.

## Materials and Methods

*Tumor samples*

Sixty-one breast tumor and paired normal breast tissues were collected from the National Cancer Institute, Bangkok, Thailand, during the period 2007-2011. This study was approved by the Institutional Review Board (IRB) of the National Cancer Institute, Bangkok, Thailand. Patients who had not received chemotherapy or radiotherapy were recruited into this study. Tissue samples were snap-frozen in liquid nitrogen and kept at -80°C until used. Clinicopathological data such as age at diagnosis, tumor size, histological grade, lymph-node status, number of lymph nodes, tumor stage, hormone receptor status and HER2, triple-negative tumor (ER-, PR- and HER2-), chemotherapy treatment (anthracycline and anthracycline+taxane), distant metastasis (bone, lung and liver) and follow-up over 13 years, were collected from medical records.

*DNA extraction and sodium bisulfite treatment*

Sixty-one breast tumors were extracted by proteinase K digestion and salting-out method (Miller et al., 1988). Sodium bisulfite conversion of DNA samples was performed using an EZ DNA Methylation Gold kit (Zymo Research, Orange, CA). One μg of extracted DNA was treated with sodium bisulfite, following the manufacturer’s instruction manual. The converted DNA was eluted in a total volume of 25 μl and stored at -20 °C until use.

*RNA Isolation and cDNA synthesis*

Fifty-one breast tumors and their corresponding normal breast tissues was extracted for total RNA by using Trizol re-agent, according to the instruction protocol (Invitrogen, Carlsbad, CA, USA). mRNA was purified by Oligotex mRNA purification kit (QIAGEN, Gmbh, Germany) and reverse-transcribed using the iScriptTM Select cDNA Synthesis Kit (Bio-Rad Laboratories, Inc., Hercules, CA) for reverse transcription-polymerase chain reaction (RT-PCR) (Invitrogen, Carlsbad, CA, USA).

*Methylation-specific PCR*

**Figure 1:**
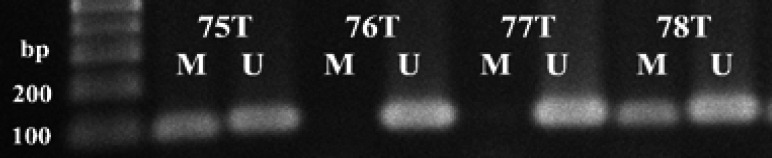
APC Promoter Hypermethylation Status in Breast-Tumor (T) Samples by Methylation-Specific PCR were Detected by Agarose Gel and Visualized by Ethidium Bromide Staining. M, methylated sequence (98 bp); U, unmethylated sequence (108 bp); bp = basepair

The APC methylation status in sixty-one breast tumors was performed by methylation specific-PCR on sodium bisulfite treated DNA. The methylated and unmethylated primers were FM- APC-5’TAT TGC GGA GTG CGG GTC-3’; RM-APC-5’TCG ACG AAC TCC CGA CGA-3’; FU-APC- 5′-GTG TTT TAT TGT GGA GTG TGG GTT-3′; RU-APC-5′-CCA ATC AAC AAA CTC CCA ACA A-3′ (Esteller et al., 2000). The reactions were conducted in a total volume of 25 μl, containing 100 ng of bisulfite-treated DNA, 1X PCR buffer, 0.2 mM of each dNTP, 2.5 mM MgCl_2_, 0.4 μM of forward and reverse primers, 0.5X GC-rich solution and 1 unit of FastStart TaqDNA Polymerase (Roche Diagnostics, Mannheim, Germany). Reaction mixtures were hot-started at 95°C for 5 min. Amplification was performed in a Mastercycler gradient (Eppendorf) for 30 cycles (1 min at 95°C, 30 sec at 59°C (methylated sequence) and 60°C (unmethylated sequence) and 30 sec at 72°C, followed by a final extension of 5 min at 72°C. 25 microliters of PCR product were electrophoresed in 1.5% agarose gel, stained with ethidium bromide, and photographed under UV light (Figure 1.). 

*APC mRNA expression level analysis by quantitative real-time reverse transcription-PCR*

Altered APC mRNA expression levels were analyzed by LightCycler Instrument (Roche Applied Science). The reaction mixture was 20 ng of template cDNA, 1x LightCycler FastStart DNA Master SYBR Green I (Roche Applied Science, Germany), 4 mM MgCl2 and 0.5 μM forward and reverse primers in a final volume of 10 μl. The primer sequences were designed by Primer3 program, forward F-APC (5’- TATCCATGCGACAGTCTGGA-3’) and reverse R-APC (5’-CCACTCCCAACAGGTTTC AC-3’. β-globin housekeeping gene was used as an endogenous reference to obtain relative expression values. PCR was started at 95°C for 5 min (to activate the FastStart Taq), followed by 40-cycle amplification (95°C for 10 s, 62°C for 30 s, and 72°C for 30 s). After the PCR, each amplification reaction was checked using a dissociation curve. PCR product purity was checked by 1.5% agarose gel electrophoresis, stained with ethidium bromide, and photographed under UV light. Relative gene expression values were computed, as previously described by Livak and Schmittgen (2001). The cutoff values for gene expression were adopted from median expression levels. Tumor gene expression <0.3-fold was evaluated as reduced-expression for APC.

*Statistical analysis*

The association between APC hypermethylation, APC mRNA expression level, and clinicopathological characteristics was examined statistically by a chi-square test, while survival was analyzed for patients who were followed up over 13 years, or until death, after surgery. The Kaplan-Meier method and Log rank test, were used to analyze between APC methylation and unmethylation groups. The Cox regression method was utilized to assess the prognostic effect for aberrant APC gene methylation on breast cancer patient survival. A P value < 0.05 was considered a significant association.

## Results

*Methylation analysis*

Sixty-one breast carcinomas were examined for APC methylation status by methylation-specific PCR. The results found 27 of 61 (44%) tumor samples were positive for methylation status (Figure 1). A statistically significant correlation was observed between APC hypermethylation and chemotherapy treatment (anthracycline and anthracycline+taxane) (OR= 6.9, 95%CI=1.5-31.01, P = 0.01), and distant metastasis (OR = 5.52, 95%CI = 1.27-24.08, P = 0.04), respectively, while there was no association between APC hypermethylation status and APC mRNA reduced-expression as Table 1. 

*APC mRNA gene expression analysis*

This study we also analyzed altered APC mRNA expression level in fifty-one breast tumors and paired normal breast tissues by quantitative real-time reverse transcription-PCR. Our findings show that there was no association between APC mRNA expression and clinicopathological parameters including survival as shown in Table 2.

*Survival of breast cancer patients*

**Figure 2:**
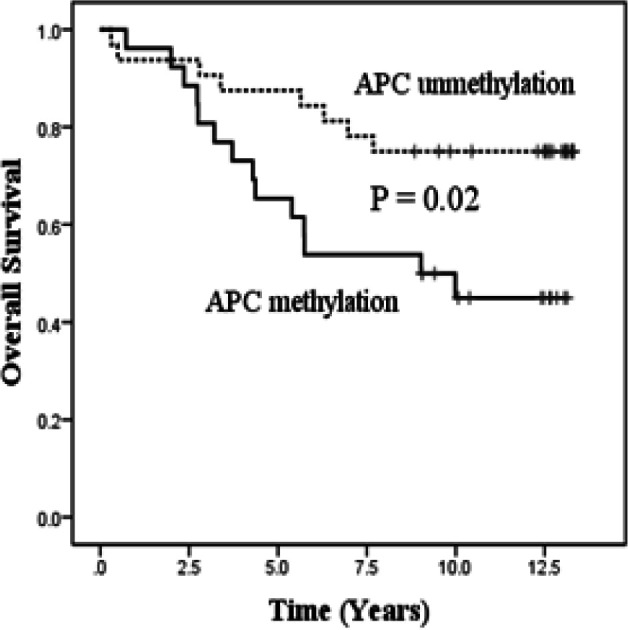
Kaplan-Meier Analysis for Overall Survival of Breast Cancer Patients and Log Rank Test was Used to Compare between APC Methylation and Those without (P=0.02)

Overall survival analysis was determined by the Kaplan-Meier survival curve and the Log rank test was used to compare the survival time between methylated and unmethylated patients. The data summarizes significant correlation between APC methylated and survival. The breast cancer patients were found APC methylated of promoter region reduced the survival period than APC unmethylated status (8.4 and 11.0 years respectively, P = 0.02, Figure 2). Furthermore, Multivariate Cox regression analysis revealed APC hypermethylation was an independent prognostic factor that affect breast cancer survival (HR =3.57, 95%CI=1.27-10.01, P=0.016), see Table 3).

**Table 1:** Association between APC Hypermethylation Status and Clinicopathological Variables in Sixty-One Breast Cancer Patients

Parameter	No.	APC methylation		Odds ratio, (95%CI)	P
		U	M		
		n (%)	n (%)		
Age				1.79, (0.64-4.96)	0.31
≤50	32	20 (62.5)	12 (37.5)		
>50	29	14 (48.3)	15 (51.7)		
Tumor size(cm)				1.13, (0.45-3.79)	0.79
≤3	37	21 (56.8)	16 (43.2)		
>3	22	11 (50.0)	11 (50.0)		
Histologic grade				2.22, (0.77-6.44)	1.85
I+II	33	21(63.6)	12 (36.4)		
III	25	11(44.0)	14 (56.0)		
Tumor stage				1.92, (0.69-5.39)	0.30
I, IIA, IIB	32	20 (62.5)	12 (37.5)		
IIIA, IIIB	28	13 (46.4)	15 (53.6)		
Lymph-node status				1.87, (0.64-5.45)	0.30
Negative	23	15 (65.2)	8 (34.8)		
Positive	38	19 (50.0)	19 (50.0)		
Lymph nodes (no.)				2.58, (0.88-7.54)	0.11
0-3 positive	39	25 (64.1)	14 (35.9)		
>3 positive	22	9 (40.9)	13 (59.1)		
Immunohistochemical					
ER status				1.54, (0.52-4.58)	0.58
Negative	21	13 (61.9)	8 (38.1)		
Positive (1+,2+,3+)	37	19 (51.4)	18 (48.6)		
PgR status				1.81, (0.63-5.19)	0.30
Negative	27	17 (63.0)	10 (37)		
Positive (1+,2+,3+)	31	15 (48.4)	16 (51.6)		
HER2 status				1.71, (0.45-6.41)	0.51
Negative	46	27 (58.7)	19 (41.3)		
Positive (1+,2+,3+)	11	5 (45.5)	5 (54.5)		
Triple negative tumor				0.39, (0.11-1.33)	0.15
ER,PR,HER2 positive	39	18 (46.2)	21 (53.8)		
ER,PR, HER2 negative	16	11 (68.8)	5 (31.3)		
APC reduced-expression				0.86, (0.18-4.13)	1.00
No	39	23 (59.0)	16 (41.0)		
Yes	8	5 (62.5)	3 (37.5)		
Chemotherapy treatment					
Antracycline	33	23 (69.7)	10 (30.3)	6.90, (1.5-31.01)	0.01*
Antracycline+Taxane	12	3 (25.0)	9 (75.0)		
Distant metastasis					
No	43	29 (67.4)	14 (32.6)	5.52, (1.27-24.08)	0.04*
Yes	11	3 (27.3)	8 (72.7)		

CI, confidence interval; M, methylated allele; U, unmethylated allele; *, statistically significant correlation

**Table 2:** Association between APC mRNA Reduced-Expression and Clinicopathological Parameters in Fifty-One Breast Cancer Patients

Parameter	No.	APC reduced-expression		Odds ratio, (95%CI)	P
		APC-	APC+		
		n (%)	n (%)		
Age				1.05, (0.23-4.74)	1.00
≤50	26	22 (84.6)	4 (15.4)		
>50	25	21 (84.0)	4 (16.0)		
Tumor size(cm)				0.25, (0.03-2.22)	0.24
≤3	31	25 (80.6)	6 (19.4)		
>3	18	17 (94.4)	1 (5.6)		
Histologic grade				1.02, (0.20-5.14)	1.00
I+II	27	23 (85.2)	4 (14.8)		
III	20	17 (85.0)	3 (15.0)		
Tumor stage				1.28, (0.28-5.83)	1.00
I, IIA, IIB	27	23 (85.2)	4 (14.8)		
IIIA, IIIB	22	18 (81.8)	4 (18.2)		
Lymph-node status				0.98, (0.21-4.70)	1.00
Negative	19	16 (84.2)	3 (15.8)		
Positive	32	27 (84.4)	5 (15.6)		
Lymph nodes (no.)				0.56, (0.10-3.13)	0.70
0-3 positive	33	27 (81.8)	6 (18.2)		
>3 positive	18	16 (88.9)	2 (11.1)		
Immunohistochemical					
ER status				0.34, (0.07-1.72)	0.23
Negative	17	13 (76.5)	4 (23.5)		
Positive (1+,2+,3+)	32	29 (90.6)	3 (9.40)		
PgR status				0.56, (0.11-2.83)	0.68
Negative	22	18 (81.8)	4 (18.2)		
Positive (1+,2+,3+)	27	24 (88.9)	3 (11.1)		
HER2 status				1.71, (0.45-6.41)	0.57
Negative	41	34 (82.9)	7 (17.1)		
Positive (1+,2+,3+)	7	7 (100)	0		
Triple negative tumor				6.00, (0.95-37.86)	0.06
ER,PR,HER2 positive	32	30 (93.8)	2 (6.3)		
ER,PR, HER2 negative	14	10 (71.4)	4 (28.6)		
Chemotherapy treatment				2.80, (0.03-2.71)	0.38
Antracycline	5	17 (73.9)	6 (26.1)		
Antracycline+Taxane	31	10 (90.9)	1 (9.1)		
Distant metastasis					
No	35	29 (82.9)	6 (17.1)	5.52, (1.27-24.08)	1.00
Yes	9	8 (88.9)	1 (11.1)		

CI, confidence interval; -, no reduced-expression; +, reduced-expression

**Table 3:** Multivariate Cox Regression Method of Prognostic Biomarkers for Survival of Breast Cancer Patients

Parameter	HR	95%CI	P
Tumor size (cm); >3 vs ≤3	1.33	0.47-3.75	0.59
Lymph nodes (no.); >3 vs ≤3	1.87	0.44-7.92	0.40
Tumor stage; IIIA, IIIB vs I, IIA, IIB	1.64	0.39-6.81	0.50
APC methylation status; methylated allele vs unmethylated allele	3.57	1.27-10.01	0.016*
APC reduced-expression; positive vs negative	0.75	0.13-4.55	0.76

HR, hazard ratios; CI, confidence interval; *, statistically significant correlation

## Discussion

Several studies have reported that APC promoter hypermethylation are involved in carcinogenesis (Esteller et al., 2000; Han et al., 2018). Especially in breast cancer, It has been indicated that the reduction of APC gene expression is induced by CpG-island hypermethylation, observed in up to 70% of breast cancers (Stefanski et al., 2019). In the present study, we observed the frequency of APC CpG-island hypermethylation of breast-cancer samples, and found that 44% of patients showed promoter hypermethylation.

This study, APC hypermethylation was found to be significantly associated with a group of patients who received adjuvant anthracycline plus taxane-based chemotherpy (P=0.01). Recently, DNA methylation have demonstrated as a potential biomarker for decision-making in the treatment of cancer patients (Duffy et al., 2009; Stefansson et al., 2013). However, this is the first research for demonstrating APC methylation status correlated with chemotherapy treatment in breast cancer. Indicating that determination of the APC promoter hypermethylation status may serve as a predictive marker for therapy monitoring and prediction of therapy response in breast cancer patients. 

Furthermore, our analysis also show that APC methylation was related to metastasis breast cancer case (P=0.04). In accordance with previous report of Debouki-Joudi et al., (2014) demonstrated aberrant methylation of APC was associated with distant metastasis (P= 0.031) in familial breast cancer. Chen et al., (2005) revealed that APC promoter hypermethylation was found in CRC hepatic metastasis. Moreover, hypermethylation of Cyclin D2, RAR-beta, Twist, RASSF1A, and HIN-1 genes was significantly increase in distant metastases compared with their primary site of breast cancer (Mehrotra et al., 2004). This suggested APC methylation may be involved with a biologically aggressive phenotype and play a role in a progression among breast-cancer patients. Two major mechanisms that contributed to metastasis, one is APC methylation frequenly occurs early in breast cancer and increases during disease progression and the other one is that ‘de novo’ occur in the metastatic cancer cells even in lacking of methylation in the primary cancer cells (Chen et al., 2005). This information that may be important to the biology of distant metastasis and that may assist in the design of therapeutic modalities (Mehrotra et al., 2004).

We also studied APC mRNA expression and results showed that there was no association between APC promoter hypermethylation and APC reduced-expression. That correlated with previous reports such as Van der Auwera et al., (2008) who reported, there was no significant correlation between APC methylation and APC mRNA or protein expression levels in breast cancer phenotypes as well as in colo-rectal cancer metastasis. Lacking the correlation between APC methylation and APC reduced-expression could be possible due to the fact that only one allele was methylated, allowing expression from the unmethylated allele (Chen et al., 2005). Therefore, hypermethylation was not the sole mechanism that affected APC expression in breast cancer. Several mechanisms affect APC expression in breast cancer (Turker et al., 2002). Such as gene mutations or allelic losses that may affect APC gene expression (Van der Auwera et al., 2008).

Moreover, our data showed that loss of APC function from promoter methylation status is trends to be aggressive behavior of cancer cell. Recently, Univariate analysis of Kaplan Meier analyses was used to evaluated the correlations between APC methylated status and patient’s survival times. Our results suggested breast cancer patients who had APC hypermethylation status shown a wores prognosis when comparing with unmethylated group (P=0.02). Additionally, Multivaritae analysis by Cox regesion model was utilize to estimate which the prognostic variables may affect survival of breast cancer patients. Analyses were adjusted for tumor size, number of lymph nodes, tumor stage, APC reduced-expression and APC methylation status, the findings revealed APC methylation status shown as an independent prognostic factor that affect survival (HR =3.57, 95%CI=1.27-10.01, P=0.016). This study indicating that APC methylation status is a risk variable that affect on survival of breast cancer patients. Sevral previous studies suggested APC methylation was associated with survival of breast cancer in Tunisian patients (p = 0.046) (Debouki-Joudi et al., 2017). DNA methylation of APC in serum of early breast cancer patients who had not undergone adjuvant systemic treatment appeared to be an independent prognostic marker for overall survival (Muller et al., 2003). The tumor suppressor APC in breast cancer, methylation frequency has been correlated with a poor prognosis (Virmani et al., 2001; Sarrio et al., 2003). APC hypermethylation of high to moderate differentiation prostate cancer patients was shown associated with poor survival and patients with an unmethylated APC gene had better survival in metastatic CRC (Richiardi et al., 2009; Matthaios et al., 2016).

In conclusion, it was found that 44% of Thai breast cancers had APC promoter hypermethylation. This hypermethylation is associated with chemotherapy treatment, distant metastasis and overall survival of patients. The APC promoter hypermethylation is an aggressive biological phenotype in breast cancer and may be used as a worse case prognosis in breast cancer patients.
